# Embryology of the fat‐tailed dunnart ( *Sminthopsis crassicaudata* ): A marsupial model for comparative mammalian developmental and evolutionary biology

**DOI:** 10.1002/dvdy.711

**Published:** 2024-05-09

**Authors:** Axel H. Newton, Jennifer C. Hutchison, Ella R. Farley, Emily L. Scicluna, Neil A. Youngson, Jun Liu, Brandon R. Menzies, Thomas B. Hildebrandt, Ben M. Lawrence, Angus H. W. Sutherland, David L. Potter, Gerard A. Tarulli, Lynne Selwood, Stephen Frankenberg, Sara Ord, Andrew J. Pask

**Affiliations:** ^1^ TIGRR Lab, The School of BioSciences University of Melbourne Melbourne Victoria Australia; ^2^ Department of Environment and Genetics, and Research Centre for Future Landscapes, School of Life Sciences La Trobe University Melbourne Victoria Australia; ^3^ Leibniz Institute for Zoo and Wildlife Research (IZW) in the Forschungsverbund Berlin eV, Reproduction Management Alfred‐Kowalke‐Straße Berlin Germany; ^4^ Colossal BioSciences Dallas Texas USA

**Keywords:** craniofacial, heterochrony, lateral plate, limb, marsupial, neural crest, patterning

## Abstract

**Background:**

Marsupials are a diverse and unique group of mammals, but remain underutilized in developmental biology studies, hindering our understanding of mammalian diversity. This study focuses on establishing the fat‐tailed dunnart (*Sminthopsis crassicaudata*) as an emerging laboratory model, providing reproductive monitoring methods and a detailed atlas of its embryonic development.

**Results:**

We monitored the reproductive cycles of female dunnarts and established methods to confirm pregnancy and generate timed embryos. With this, we characterized dunnart embryo development from cleavage to birth, and provided detailed descriptions of its organogenesis and heterochronic growth patterns. Drawing stage‐matched comparisons with other species, we highlight the dunnarts accelerated craniofacial and limb development, characteristic of marsupials.

**Conclusions:**

The fat‐tailed dunnart is an exceptional marsupial model for developmental studies, where our detailed practices for reproductive monitoring and embryo collection enhance its accessibility in other laboratories. The accelerated developmental patterns observed in the Dunnart provide a valuable system for investigating molecular mechanisms underlying heterochrony. This study not only contributes to our understanding of marsupial development but also equips the scientific community with new resources for addressing biodiversity challenges and developing effective conservation strategies in marsupials.

## Key Findings

- Methods for husbandry, detection of pregnancy and timed embryo collection in a laboratory colony of fat‐tailed dunnarts.
- Full charactertization of dunnart embryogenesis from cleavage to birth.
- Generate stage‐matched embryonic comparisons between the dunnart and other amniotes.

## 1 INTRODUCTION

Mammals are a highly diverse and successful lineage of vertebrates, with over 6400 extant species across three clades—the Prototheria, Metatheria, and Eutheria, also known as monotremes, marsupials and placentals, respectively. Despite sharing defining mammalian characteristics, each clade has evolved their own distinct reproductive strategies and modes of development, producing unique patterns of development. While the embryonic growth and development of several groups of placentals (including, but not limited to species in references^1^, ^2^, ^3^, ^4^, ^5^, ^6^), and the limited diversity of monotremes^5^, ^7^ has been documented, studies detailing the embryonic growth and development of marsupials are significantly lagging. The small handful of marsupial embryological studies emphasize the considerable gap in our understanding of marsupial biology, and their larger contributions to mammalian development. This is especially concerning, as global rates of biodiversity loss are increasing at a dramatic rate, with ~70% population declines since 1970 and approximately 40% of Australian marsupials now considered Threatened.^8^, ^9^ With anthropogenic habitat loss, predation by invasive species, and climate change‐related events posing continued threats to biodiversity,^10^, ^11^ there is a pressing need to improve our understanding of the reproductive and developmental biology of the world's diverse marsupial fauna, to ensure the ongoing development of novel conservation strategies.

Placental and marsupial (Therian) mammals last shared a common ancestor ~160 million years ago,^12^ and have evolved unique developmental trajectories in response to their distinct reproductive strategies. Eutherian mammals typically give birth to well‐developed young after an extended gestation, with extensive in‐utero nutritional support provided through an invasive placenta. In contrast, marsupials give birth to highly underdeveloped (altricial) young after a relatively short gestation, that is predominantly reliant on histotrophic nutrition and a relatively superficial placenta.^13^ At birth, the altricial marsupial neonate is required to crawl into the mother's pouch and attach to the teat to continue development through an extended period of lactation.^14^, ^15^ This unique mode of marsupial reproduction has enforced constraints on their development, where marsupials have evolved a comparatively rapid organogenesis including altered timing of key developmental events in utero, known as heterochrony.^16^, ^17^, ^18^, ^19^, ^20^, ^21^ This intraspecific heterochrony is especially seen by accelerated development of the orofacial complex and forelimbs^20^, ^22^ to meet the functional demands of crawling to the pouch and sucking.^23^, ^24^ Whereas at birth the hindlimbs, and neurological system (eyes, brain) are rudimentary,^25^, ^26^, ^27^ with the exception of the neural somatosensory system, allowing the newborn to navigate its way towards the pouch.^15^ These heterochronic patterns of development, notably the accelerated orofacial and limb development, further extend between marsupials,^28^ which possess additional interspecific differences in timing (classed from G1 ultra‐altricial to G3 or less altricial^29^), and even more broadly across other studied vertebrates, including chicken or mouse.^16^, ^17^, ^20^, ^21^, ^30^, ^31^, ^32^ Therefore, contrasting patterns of developmental heterochrony between marsupials and other vertebrates offer fascinating insights into the cellular and genetic regulation of homologous anatomical features between species.

In order to address these questions, suitable laboratory models are required to generate stage‐matched marsupial embryos. Previous decades of research have seen investigations on the embryology of American Virginia opossum (*Didelphis virginiana*)^33^ and gray short‐tailed opossum (*Monodelphis domestica*),^34^ as well as Australian tammar wallaby (*Notamacropus eugenii*),^31^, ^35^ agile antechinus (*Antechinus agilis*),^36^ and *s*tripe‐faced dunnart (*Sminthopsis macroura*),^37^ providing unique insights into distinct aspects of marsupial developmental biology. However, of these only *Monodelphis* has persisted as an amenable laboratory‐based colony for developmental and genetic studies, such as a characterized genome,^38^ transcriptomics,^39^ and described methods for genetic manipulation.^40^ Given the ~80‐million‐year divergence between North American and Australian marsupials, a laboratory‐based Australian marsupial model with similarly easy husbandry, year‐round breeding, and experimental manipulation is still needed for a more complete understanding of several aspects of Australian marsupial biology, and the broader mechanisms underlying mammalian heterochrony.

In an effort to bridge this gap, the fat‐tailed dunnart (*Sminthopsis crassicaudata*; hereafter referred to as the dunnart) is being established as an Australian marsupial model for comparative developmental and reproductive biology.^41^ The dunnart has a gestation of ~14 days,^42^, ^43^, ^44^, ^45^ and several aspects of its embryonic development have been described; including oocyte maturation, fertilization, embryo transfer, development of cleavage stages in vitro and placentation^42^, ^45^, ^46^, ^47^; as well as high‐resolution characterization of its postnatal development, including targeted genetic manipulation of pouch young.^25^, ^26^, ^48^ Furthermore, new efforts are being made to make the dunnart the gold‐standard laboratory marsupial, including a chromosome‐scale genome assembly.^41^ However, despite these studies and resources, a detailed characterization of the embryonic growth, development, and organogenesis of this laboratory marsupial are lacking. This is an essential resource for any laboratory model and is necessary to demonstrate its suitability for comparative embryological and developmental investigations between mammals. Furthermore, being recently reclassified and recognized as a Threatened species “Vulnerable” to extinction (Department of Energy, Environment and Climate Action [2023]. Flora and Fauna Guarantee Act 1988 Threatened List),^49^ establishing the fat‐tailed dunnart as a laboratory model provides a direct conduit to research into management and restoration of vulnerable species.

In this study, we present an atlas of embryo development of the fat‐tailed dunnart, complementing existing early and post‐natal staging series.^25^, ^26^ To enhance the accessibility and enable other laboratories to establish this marsupial model, we provide detailed methods for reproductive monitoring to detect and confirm pregnancy, timed embryo collection, and a companion study detailing best practices for dunnart housing, handling, maintenance, and husbandry.^50^ We additionally generate embryological comparisons with other vertebrate model species, including human, mouse, opossum, and chicken, to create a unified stage‐matched atlas of development, highlighting the onset of heterochronic developmental patterns unique to marsupials. Through these descriptions and comparisons, this series provides a detailed overview of the reproductive and developmental biology of an Australian marsupial and enhances our understanding of mammalian development and its diversity.

## 2 RESULTS

### 2.1 Experimental monitoring and defining the Dunnart reproductive cycle

#### 2.1.1 Weight fluctuations are a strong indicator of ovulation and pregnancy

Between May to December, daily body weight fluctuations were monitored across 71 female dunnarts which were used to generate pouch young, or embryos of specific developmental stages. Monitored females which gave birth were used to initially establish the weight profile, and then additional monitored and collected females carrying embryos from the primitive streak stage (stage 19) were used to further calibrate the weight profile. The average body weight of a reproductively active female, at any stage of their cycle, was 16.28 ± 2.09 g (Table S1). The Dunnart reproductive cycle is ~31 days consisting of a ~15‐day anoestrous, ~3‐day estrous (including ovulation), and ~14‐day diestrus period, after which pregnant animals give birth. Using these stages, we established a growth curve to predict the stage of pregnancy for timed embryo collection.

**FIGURE 1:**
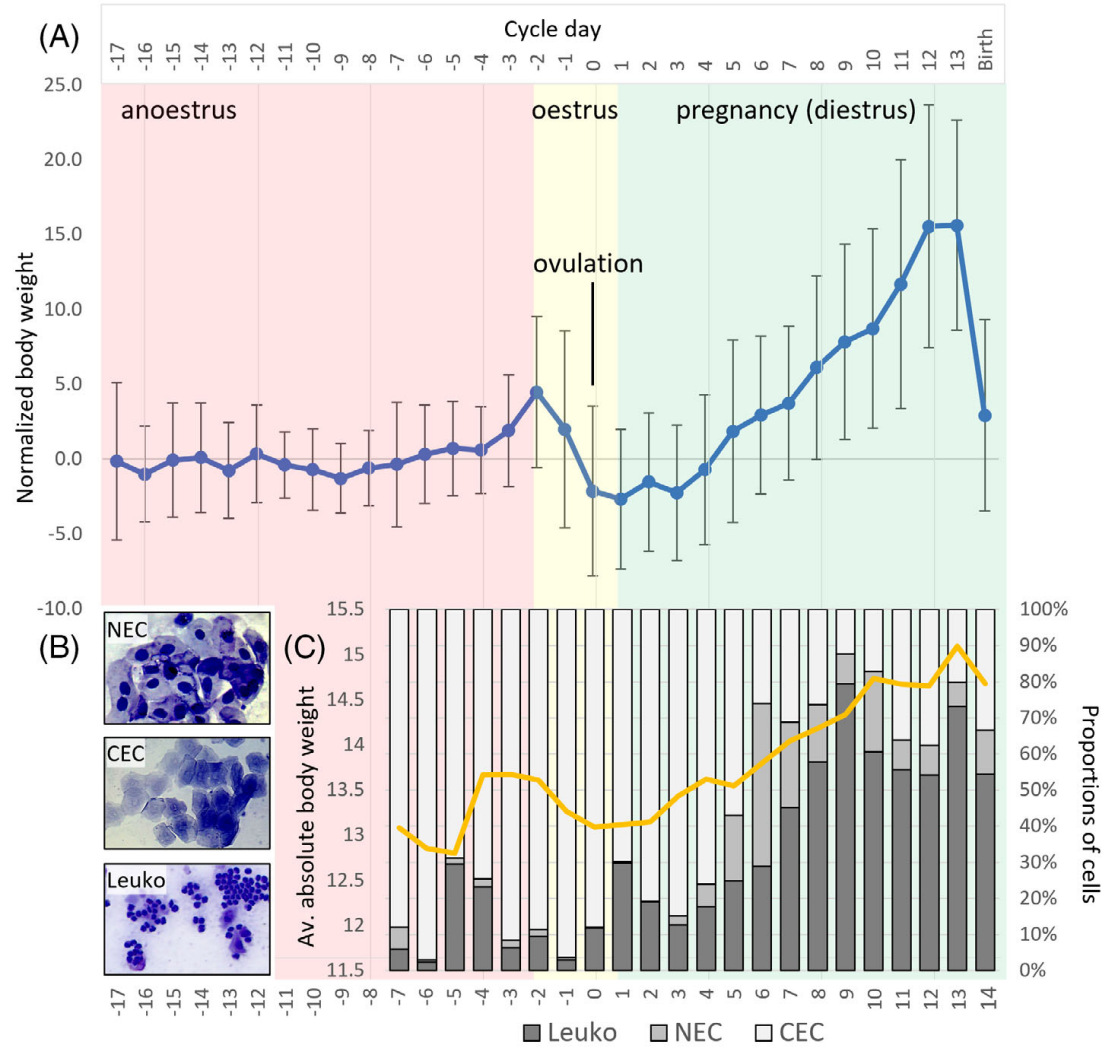
Monitoring methods for detection of dunnart estrus and pregnancy. (A) Average weight changes across the 31‐day reproductive cycle (grams, g). Weights were monitored daily, and the average base weight was calculated per animal to generate normalized weight change profiles across pregnancy. Ovulation was marked by a sharp decrease in weight, followed by a gradual increase of ~3 g over pregnancy before a sharp decrease during birth. Non‐pregnant females showed a similar pattern, albeit with a lesser weight increase of 1–2 g, which were easily distinguishable by ~Day 10 post‐ovulation. Each datapoint is the mean across 37 dunnart weight profiles, and error bars represent standard deviation of the mean. (B) Cell types present in dunnart urine. Nucleated epithelial cells (NECs), cornified epithelial cells (CECs), and leukocytes (Leuko). (C) Relative proportions of cells in dunnart urine in cycling, non‐pregnant females. Anoestrus females show high proportions of CECs. Ovulation (dip in body weight) is accompanied by a spike in relative leukocyte numbers, followed by steady increase in leukocytes during diestrus.

To normalize weight fluctuations across animals of different ages and body mass, we calculated the base weight for each animal, taken as the average weight across the anoestrous period. This base weight was used to calculate daily weight changes, taken as a percentage change from the base weight, yielding a proportional daily weight change for all animals across their cycle. The average daily weight change from each animal was plotted and aligned by their ovulation dip, which was determined following two criteria: (1) the day of ovulation was first determined from females that gave birth, that is, 14 days before the presence of pouch young, and (2) stage matching embryos to that of *Monodelphis*.^34^ This revealed a consistent pattern across the ~31‐day reproductive cycle, including a weight dip indicating ovulation, progressive weight increases during pregnancy and a sharp weight decline at birth (14 days post ovulation; Figure 1A), echoing similar patterns observed in *S*. *macroura*.^37^, ^51^ Raw and transformed weight values are detailed in Table S1. Using this protocol, 71 female dunnarts at various stages of their reproductive lifecycle (i.e., virgin females, past mothers, or females post pouch young removal) were monitored and collected at specific timepoints to collect various staged embryos (Table S1). Using our method, embryos were present in 54 out of the 71 females, representing a 76% overall success rate for embryo collection.

#### 2.1.2 Urogenital lavage cytology identifies the onset of estrous and ovulation

While a sharp decrease in weight was confirmed to be a strong indicator of ovulation, determining the transition between anoestrous and estrous in a newly monitored cycling female was difficult. We therefore performed additional urine cytology on females over a span of 4 cycles (~120 days), to detect the presence and relative abundances of NECs, CECs, and leukocytes (Figure 1B), which has previously been shown to be a strong predictor of oestrus in in *A*. *agilis* ^36^ *and S*. *macroura*.^37^, ^51^ In nonpregnant females, the presence of NECS, CECs, and leukocytes changed in relative proportions across the 31‐day cycle (Figure 1C). During the anoestrus period, females showed a high abundance of CECs, with few NECs and some leukocytes. However, leading into estrous and ovulation, there was a spike in leukocytes accompanying their dip in weight (Figure 1C). Following ovulation, we observed a steady increase in leukocytes and NECs, and decrease in CECs during the diestrus period (only observed in non‐pregnant females). For females paired with a reproductively mature male, daily weight profiles, namely a sharp decrease in weight, with accompanying high proportions of CECs and subsequent increase in leukocytes, is a strong predictor of oestrus and ovulation (Figure 1). If sperm is additionally detected in the females urine, this is a strong indicator that ovulation and fertilization have occurred.

### 2.2 Reproductive tract morphology across gestation

**FIGURE 2:**
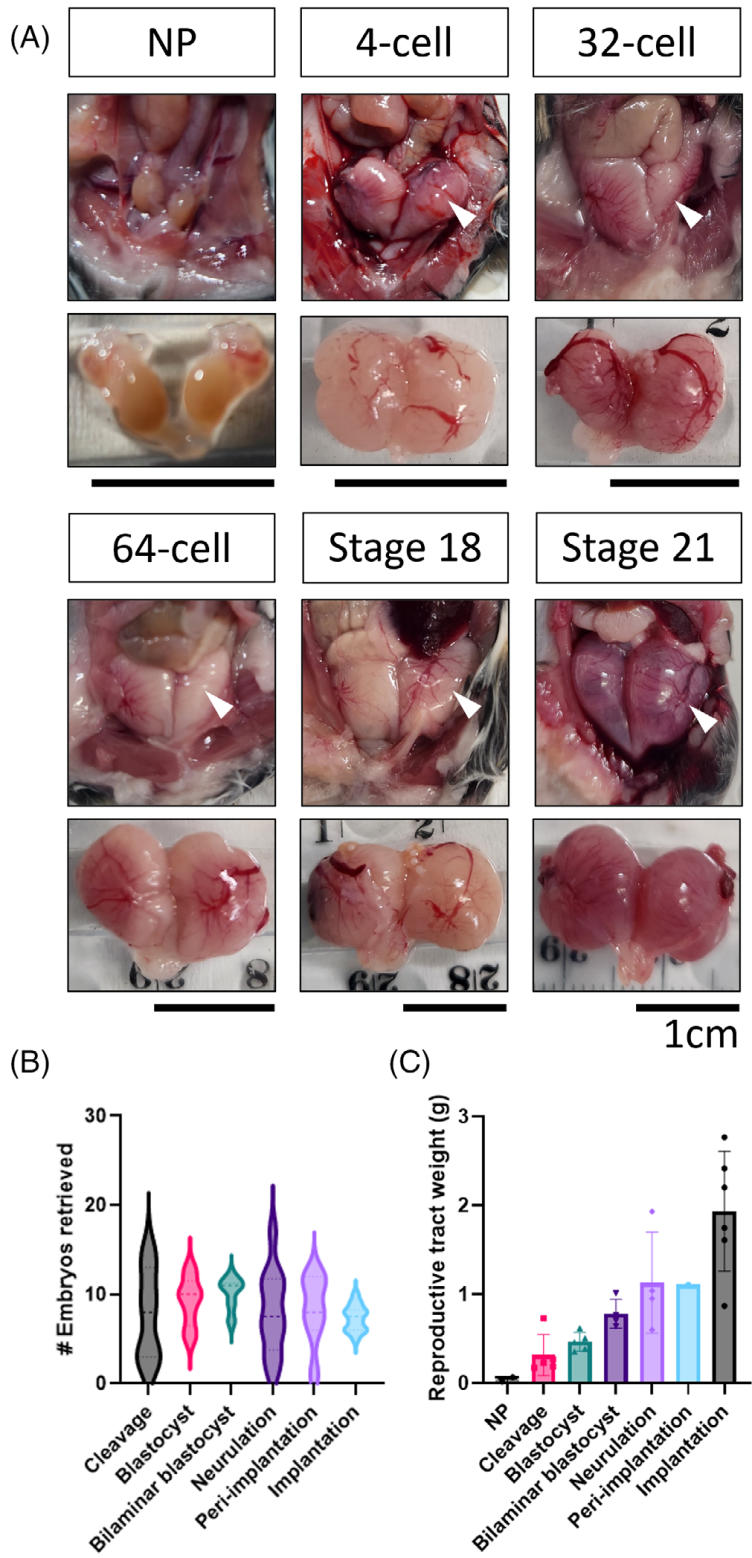
Changes to the gross physiology and morphology of the dunnart uterus during pregnancy. (A) Representative images of the nonpregnant (NP) and gestating uterus. The dunnart uterus is situated in the abdominal cavity (arrowhead) and substantially increases in size across gestation. Embryonic stage is indicated above each photograph. (B) Number of embryos retrieved from the uterus at specific time points. The number of embryos retrieved from the pregnant uterus up to neurulation ranged between 1 and 17 per animal. Between 1 and 12 peri‐implantation embryos could be retrieved, and up to 10 implantation stage embryos could be retrieved from the pregnant uterus. Violin plot shows median, and quartiles derived from ≥6 biologically independent replicates per group. (C) Weight (grams, g) of the reproductive tract (comprising ovaries, uterus, embryos, cervices) across gestation. Data shown as mean ± standard deviation, individual dot points denote independent biological replicates. Gestation stages: cleavage: embryos containing ≤16 cells; blastocyst: unilaminar blastocyst containing ≥32 cells; bilaminar blastocyst: embryo has a clear embryonic area; neurulation: stages 19–23; peri‐implantation: stages 24–27; implantation: stages 28–33. Data visualized using GraphPad Prism.

The dunnart has duplex uteri, of discoid shape (Figure 2A). Each uterus has an independent ovary, oviduct, and cervix, with the cervices joining at the urogenital sinus. The refractory uterus is small and pale (Figure 2A; NP) and is not much larger than the cervix in size. Within the peritoneum, the uteri sit behind the intestines, and are not immediately evident. They can be easily found by identifying the bladder and gently pulling forward the urogenital sinus and the attached reproductive tract. In pregnant females, embryos are found in both uteri during gestation in similar quantities, but slightly differing in their stages of development, particularly during cleavage stages. For example, a mix of 2‐ and 4‐cell embryos, 4‐ and 8‐cell embryos, and later a mix of blastocysts of varying sizes were present in the uterus. In *S*. *macroura*, ovulation occurs in a pulsatile manner over a period of a few hours, resulting in the cohort of embryos encompassing a small range of stages, a phenomenon possibly conserved in *S*. *crassicaudata*.^52^ The number of embryos retrieved from the uteri of a pregnant dunnart is variable across developmental stages (Figure 2B), with up to a maximum of 17 (mean = 8) neurula embryos retrieved from a single dunnart during this study. When collected at a stage near birth, the number of embryos retrieved from a single dunnart in this study did not exceed 10 (mean = 7), aligning with the total number of nipples present in the pouch.

During the period surrounding ovulation, the fimbria of the oviducts increase in size becomes highly vascularized, and enclose the ovary. Gestation is accompanied by alterations in the size and weight of the reproductive tract (Figure 2C). Pregnant uteri are more easily observed during dissection than their refractory counterparts, having partially displaced the intestines due to their increase in size. Uterine expansion appears to occur in two waves, reflective of the body weight increase during gestation. The first wave of expansion occurs in the first 5 days of gestation (encompassing embryonic cleavage stages), with an enlarged uterus observed by the time the embryo reaches the 4‐cell stage (Figure 2A). Further expansion occurs as the blastocyst develops through to the primitive streak stage (stage 19, Day 10 of gestation). At this stage, the embryo is still contained within a shell coat that is present from early cleavage stages and is not attached to the uterine epithelium. Secondary uterine expansion occurs following shell coat breakdown, around Day 10–11 of gestation. A significant increase in size occurs concurrently with a visually appreciable increase in maternal vascularization, evidenced by large vessels and the red tinge of the uterine tissues. During this time, the embryo emerges from the shell coat and can attach superficially to the endometrial luminal epithelium. An increasing weight of the reproductive tract and uterine contents is evident through implantation stages (Figure 2C), accounting for approximately 50% of the increasing body weight (Figure 1A). The weight of the reproductive tract presented in Figure 2C encompasses the weight of the cervices, uteri, ovaries, oviducts, and embryos.

### 2.3 Confirmation of pregnancy and fetal monitoring by abdominal ultrasound

**FIGURE 3:**
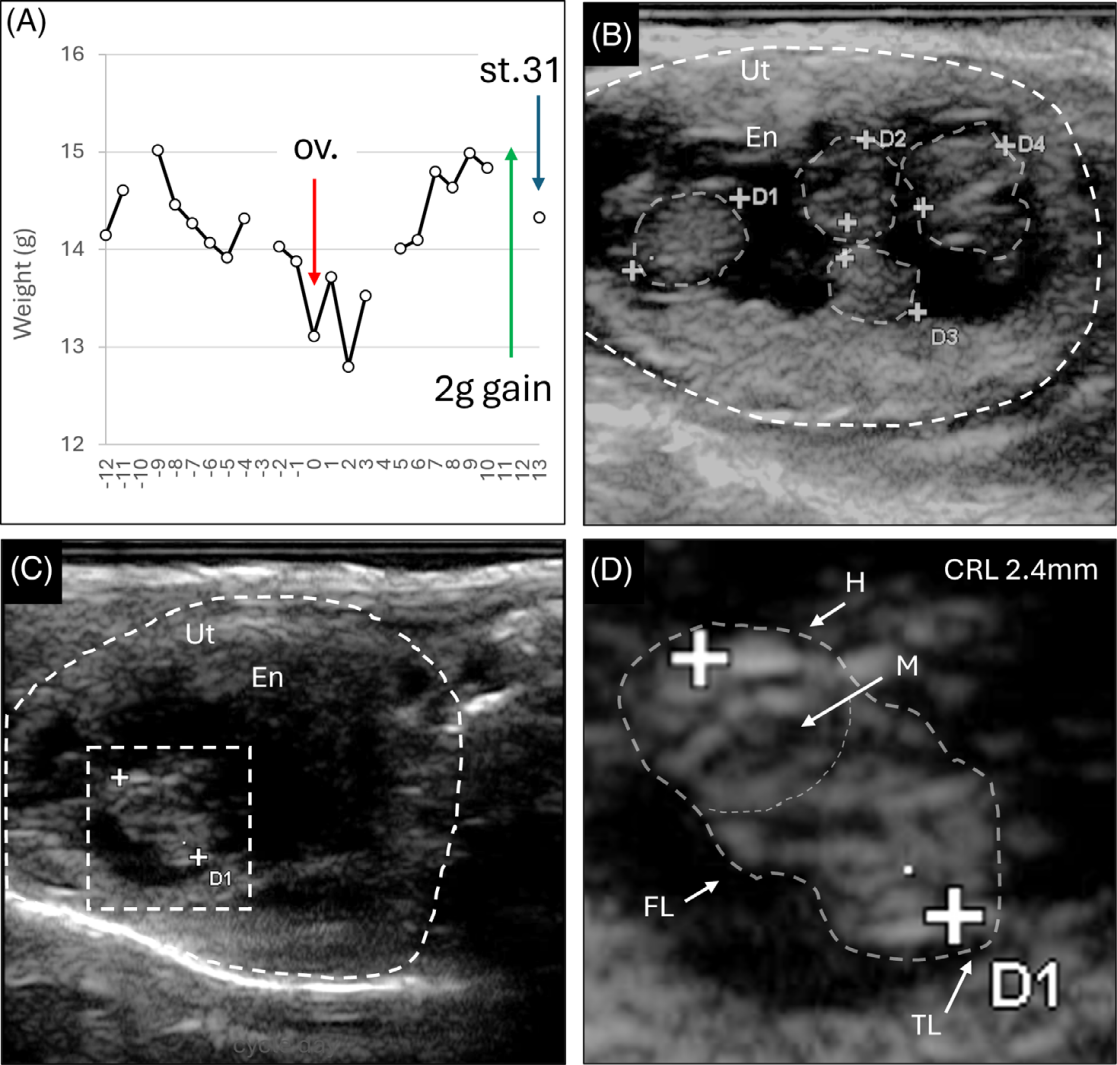
Ultrasound examination of the dunnart uterus during pregnancy. (A) Weight (g) monitoring of a female dunnart suspected to be pregnant, where examination occurred at approximately 13 days of gestation. (B) Ultrasound examination of the uteri revealed the presence of four fetuses within one uterus. (C, D) Closer examination of one fetus revealed the presence of distinct head and oral cavity (mouth) and suggested fetuses were approximately Stage 31, corresponding with the weight monitoring profile. Ut: uterus; En: endometrium; D1–4: fetuses; H: head; M: mouth; FL: forelimb; T: tail, CRL: crown‐rump length.

**FIGURE 4:**
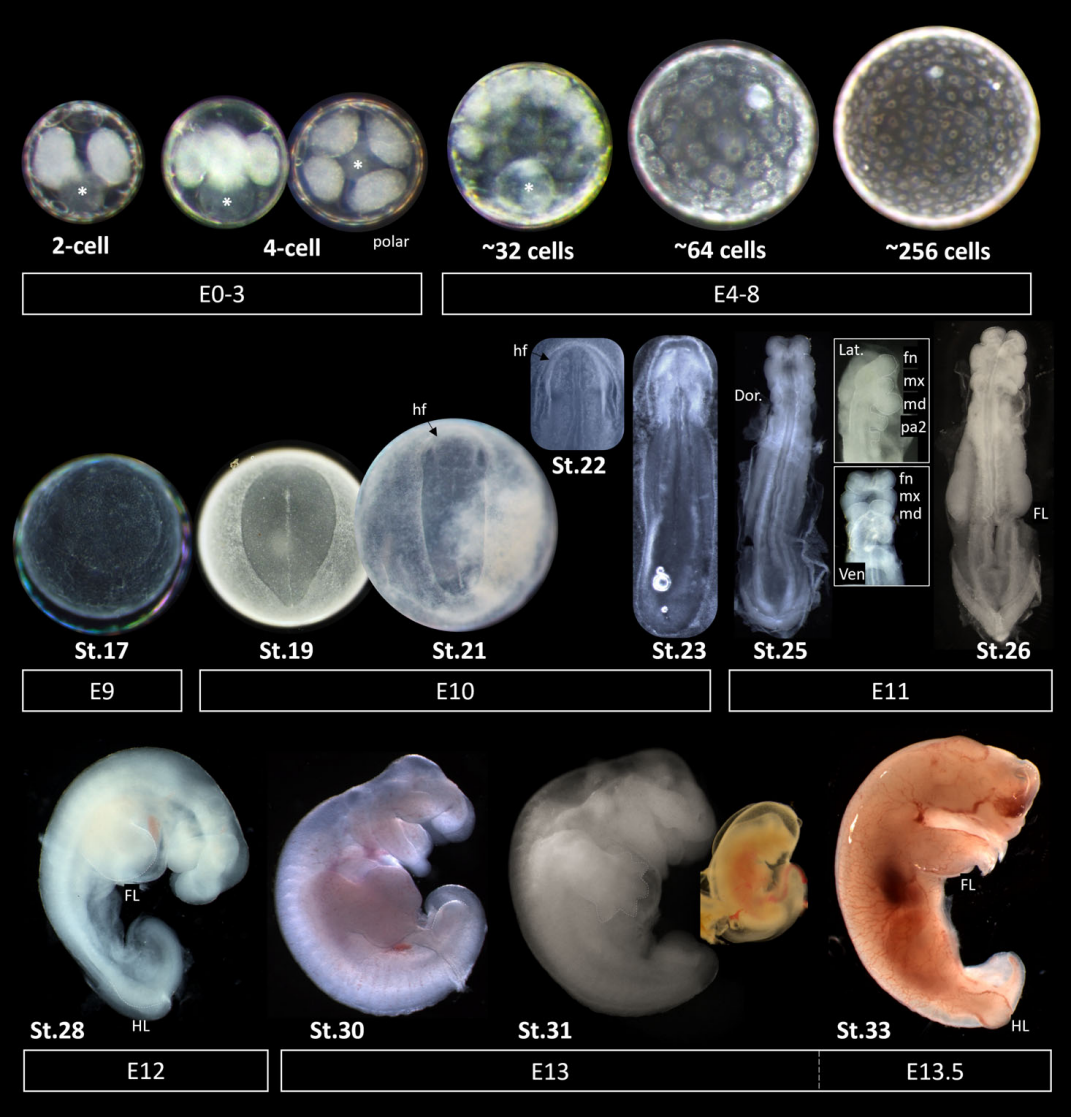
Embryonic staging series of *Sminthopsis crassicaudata*, from 2‐cell to birth. Stages were assigned based on the McCrady staging series in opossum.^33^, ^34^ Gestation and organogenesis occur over 14 days, with birth occurring typically in the morning. Cleavage occurs over a period of about 3–4 days, resulting in the formation of a unilaminar blastocyst. The blastocyst consists of a planar disc, the embryonic area, and trophoblast/trophectoderm, with the pluriblast apparent by embryonic day (E) 9 and primitive streak by E10. The headfolds form rapidly between E10 and E11. The craniofacial prominences and forelimbs undergo rapid outgrowth over the final days of gestation (E11–E13.5), while the hindlimbs are delayed. Late‐stage (E13) embryos undergo a transient 1‐day implantation, observed through the formation of an umbilicus. Embryo images are not to scale. * deutoplasm. hf: head fold; fn: frontonasal process; mx: maxillary arch; md: mandibular arch; pa2: second pharyngeal arch; FL: forelimb; HL: hindlimb; Dor: dorsal; Lat: lateral; Ven: ventral.

To explore whether Dunnart fetuses can be visualized noninvasively during pregnancy we scanned experimental animals using high‐resolution portable ultrasound. Routine use of this technique has immense benefits for ongoing colony monitoring. For example, ultrasound has been used to confirm marsupial pregnancy, minimize unnecessary euthanasia of nonpregnant females, and generate counts of embryos/fetuses in utero.^53^ We therefore performed ultrasound on an anesthetized female who was actively being weight monitored and suspected to be pregnant, at approximately 13 days of gestation (Figure 3A). This successfully revealed the presence of four fetuses within one uterus (Figure 3B) confirming pregnancy. Close examination of one of the embryos showed distinct morphological characteristics, including a forward‐facing head and visible mouth, forelimb buds, and tail (Figure 3C,D), corresponding to an approximately Stage 31 fetus (Figure 4), confirmed by embryo collection. Together, these demonstrate ultrasound as an effective tool for both confirmation of pregnancy and estimation of developmental stage in laboratory animals.

### 2.4 Embryonic growth and development in *S*. *crassicaudata*

Dunnart embryos were collected from pregnant females, as per methods detailed above, and staged based on the McCrady staging series of marsupial embryos, established in *Didelphis virginiana* ^33^ and adapted to *Monodelphis domestica*.^34^ Dunnart's development followed a similar timetable and developmental patterns to those seen in the opossum (and other marsupials), which have been described in detail previously.^20^, ^22^, ^34^, ^36^, ^37^, ^54^ However, dunnart embryogenesis was slighter faster than that seen in the opossum. Cleavage and expansion of the Dunnart blastocyst occurred between Day 1 and 10 post‐fertilization, followed by rapid organogenesis between Day 10 and Day 14. Birth occurred in the morning of Day 14 and typically persisted throughout the day.

**FIGURE 5:**
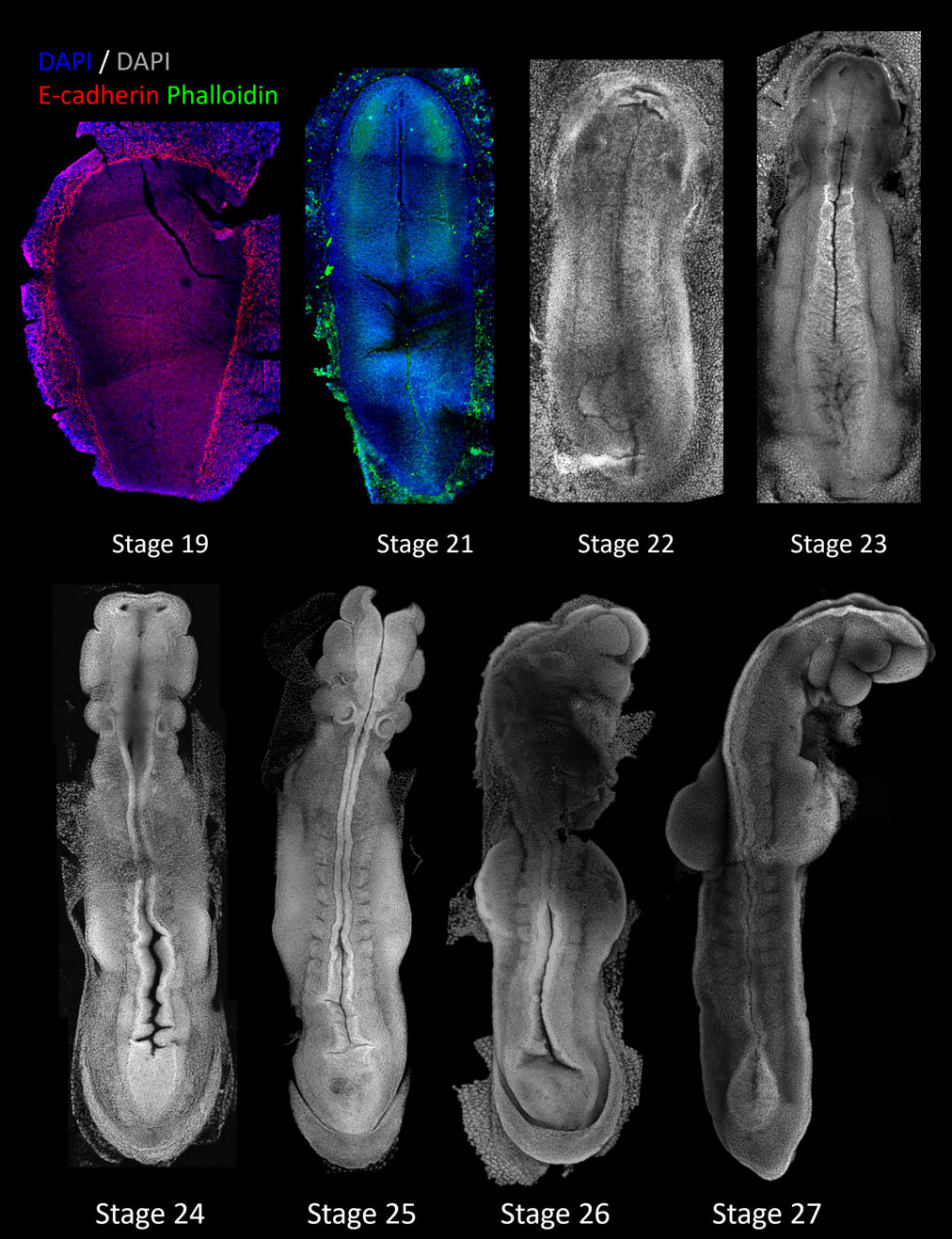
Confocal imaging of early organogenesis embryos. High‐resolution images of stage 19, 21, 22, 23, 24, and 25 embryos stained with DAPI to highlight individual nuclei. Stage 19 and 21 were co‐stained with E‐cadherin or Phalloidin (F‐Actin) to highlight embryonic features. The primitive streak is visible as a distinct line in the stage 19 trilaminar blastocyst and groove in the stage 21 neurula. Dense clusters of cells can be seen in the developing head folds on the stage 21 embryo, predicted to be early neural crest cells.^21^, ^22^ By stage 23, distinct swellings of the early facial prominences were visible, as well as the first somite pairs. By stage 24–25, several different structures are beginning to form which are easily distinguishable by high‐resolution imaging, including the developing limbs.

Organogenesis and the onset of gastrulation began on day 10 (McCrady^33^, ^34^ stage 19), through the first formation of the primitive streak and neural plate. Subsequent hours saw formation of the head folds noting development through to stage 22. On Day 11, stage 24–26 embryos were observed (we were unable to collect stage 23 which is assumed to develop overnight) marked by the onset of somitogenesis and axial elongation. By Day 12, stage 27–29 embryos had undergone cranial (cephalic) rotation including early outgrowth and patterning of the craniofacial prominences, as well as advanced forelimb formation into paddles. Day 13 saw development into early fetal stages (stage 30–33) which began to resemble pouch young, notably with clear demarcation of the facial prominences, jaws, and digits, distinct umbilical vein, and implantation into the uterus. On Day 14, birth occurred typically early in the morning and continued throughout the day, depending on litter size. Descriptions below of embryogenesis are in reference to Figures 4 and 5.

#### 2.4.1 Cleavage and blastocyst stages

The timings of cleavage and blastocyst stage embryos in the fat‐tailed dunnart were similar to those of the stripe‐faced dunnart (*S*. *macroura*).^37^, ^45^ Up to 3 days post‐ovulation, embryos retrieved from the uterus had between 2 and 8 separate but grouped cells, with an overt mucoid coat, in which sperm could still be observed. The deutoplasm can be seen at the abembryonic pole as a spherical mass (deutoplast; Figure 4, asterisk) adjacent to the ring of blastomeres. Of note, occasionally parthenogenetic embryos were observed in females that had not been mated, or whose weight increase did not follow the trend set out in Figure 1. These parthenogenetic embryos contained eight or fewer cells, in accordance with previous observations.^55^ Eight‐cell embryos contained a single ring of blastomeres, and the next cleavage division resulted in two stacked rings of eight cells. By Day 4, the embryos retrieved from the uterus contained approximately 32 cells (forming a unilaminar blastocyst) and by Day 5–6, there were between 64 and 256 cells present. In the cleavage stage embryo, cell‐zona adhesions form in preference to cell–cell adhesions, thus no compacted morula forms. From the 16‐cell stage, cell–cell contacts arise, and the blastocyst forms as a hollow sphere with a unilaminar epithelium.^56^ The unilaminar blastocyst then expands in size, reaching up to ~700 μm in diameter prior to the pluriblast becoming distinct. While at this stage the blastocyst is unilaminar, distinct cell sub‐populations are present which will give rise to the pluriblast and the trophoblast,^57^ and by Day 8–9, the embryo had formed as a distinct bilaminar disc (of epiblast and hypoblast) which lay at the embryonic pole surrounded by trophoblast/trophectoderm.

#### 2.4.2 Organogenesis

Morning collection at Day 10 post‐ovulation yielded trilaminar blastocysts embryos with an obvious primitive streak (stage 19) marking the onset of organogenesis. The embryo at this stage is pear (or pellucid)‐shaped at the embryonic pole of the blastocyst, with distinct streak and Hensen's node organizer. Afternoon embryo collections (approximately 6 h later), yielded embryos ranging from stage 21 to 23, but largely neurula stage embryos which had lost their pear shape, instead appearing elongated and thin (stage 21), but still were yet to form somites. These embryos possessed rapid and characteristic^22^ formation of distinct head folds, consisting of thickened ridges of cranial neural plate epithelium that protruded from the embryonic disc. The neural plate borders first appeared as dense regions lateral to the embryonic midline, which were packed with cells (Figure 5). Shortly thereafter (stage 22–23), the first few somite pairs were becoming visible along the length of the embryo, while the cranial headfolds formed thickened ridges and/or produced early swellings of the facial primordia.^21^, ^22^, ^28^ Notably, the formation of the neural folds and outgrowth of the frontonasal process and first pharyngeal arch appeared at an accelerated rate to that seen in other amniote embryos, marking the onset of craniofacial heterochrony which is rapid in Marsupials.^30^

Collection at 11 days post fertilization yielded early‐somite stage neurulas (stage 24–26). Embryos collected in the morning (stage 24) possessed approximately 4 somite pairs (though these were difficult to demarcate) and showed rapid, and considerable development of the head relative to the developmental stage, showing the clear formation of the frontonasal process, first pharyngeal arch (with distinct separation of the maxillary and mandibular processes) and second pharyngeal arch, despite the neural tube remaining open (Figures 4 and 5). The amnion and neural tube had merged and closed at the first 1–3 somite level, though remained open at the caudal end of the embryo. Forelimb ridges had formed from within the lateral plate mesoderm, further showing acceleration and developmental heterochrony compared with mouse and chick,^20^, ^31^ while the hindlimb ridges were yet to form. The embryonic heart tube had formed on the ventral aspect of the embryo. Collection in the afternoon on day 11 yielded stage 26 embryos which showed clear demarcation of 8–9 somite pairs, closure and fusion of the anterior neural tube, and further development of the head and limbs. The facial prominences were larger and more distinct, as well as the presence of visible otic placodes. Distinct forelimb buds are seen to protrude from the embryo, without any visible hindlimb ridges. The tail bud was formed and pointed, though the posterior neuropore remained open.

Twelve days post‐fertilization produced stage 27–29 late preimplantation embryos and early fetal development. Embryos at this stage had undergone flexion and torsion, displaying a lateral profile. Stage 28 embryos possessed large facial prominences, namely distinct and separate maxillary, and mandibular processes with signs of elongation into the jaw primordia. The externalized heart had begun beating, transporting fetal blood to the developing extremities of the embryo. The forelimb had begun to form a paddle, with constriction of the proximal limb primordia, while the hindlimb had started to form as a small ridge at the caudal end. Despite the rapid formation of the limbs from the somatic lateral plate mesoderm, the splanchnic lateral plate was yet to fuse along the embryonic ventral midline and form the digestive system. Afternoon collections (stage 29) embryos were further advanced with larger limbs and notable curvature and outgrowth of the tail bud.

Development over the next 12 h showed dramatic changes in the appearance of the fetus. Collection at 13 days post fertilization (stage 30–33) produced fetuses with well‐developed craniofacial features, including obvious upper and lower jaws, tongue, and nasal pits, whereas the eyes and fore/hindbrain were not visible or rudimentary. During these stages, the forelimbs undergo demarcation and development of the digits and claws, and the hindlimbs quickly form small paddles. The neural tube was closed along the entire axis of the embryo, and a short tail was present. The heart had internalized, and the cervical swelling—characteristic of dasyurid neonates^25^—had inflated below the head, though the body wall around the gut remained partially open. The Stage 31 fetus possessed a distinct umbilicus and signs of implantation into the maternal endometrium. Afternoon collections yielded Stage 33 late‐stage fetuses, which were considered prebirth stages. These closely resembled day 0 neonatal pouch young, where the maxilla and mandible had begun to fuse to form the oral apparatus (mouth). The forelimbs had turned to their resting position, and the digits had claws to aid in the crawl to the pouch.

### 2.5 Establishing stage‐matched embryonic comparisons with other amniotes

**FIGURE 6:**
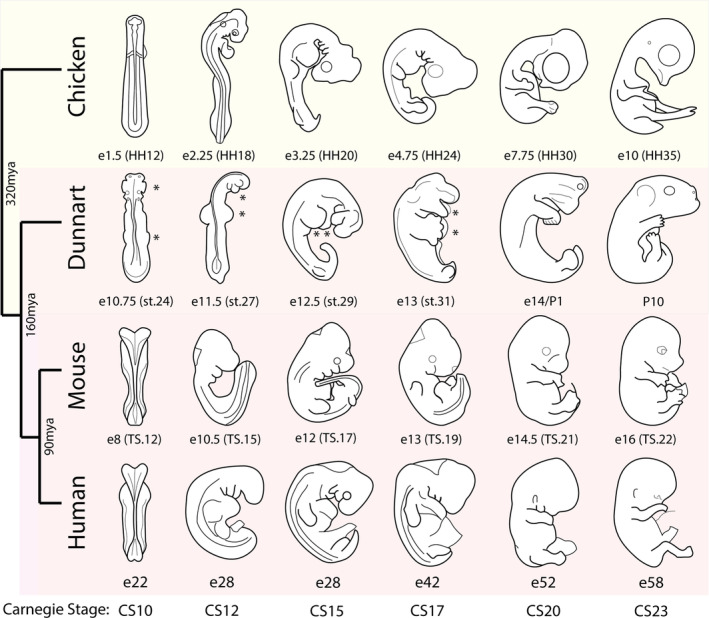
Comparative embryonic development between amniotes. Redrawn illustrations of human, mouse, dunnart, and chicken embryos, matched by Carnegie Stage (CS). CS comparisons were taken from USNW embryology, and gross morphological characteristics of dunnart embryos were aligned to CS, including the first appearance of somites, development of the heart, closure of the posterior neuropore, flexion, and torsion, and broad patterns of limb and head development. The dunnart head and limbs show developmental heterochrony, marked by an asterisk (*). Illustrations are redrawn from Halley,^58^ and comparisons are based off^25^, ^26^, ^58^, ^59^ with some modifications.

To establish timed embryological comparisons between the dunnart and other vertebrate species, we leaned on established pairwise estimations of developmental timing across mammals and birds. Developmental comparisons have been generated between mammals, including human Carnegie stages and mouse Thieler stages,^58^ as well as broader comparisons with chicken Hamburger–Hamilton stages.^59^ Furthermore, pairwise comparisons of postnatal growth and development of the dunnart have been made between mouse and human,^25^, ^26^ however, these had not been extended into pre‐natal embryonic stages. We, therefore, filled and refined these gaps, based upon the UNSW Embryology Carnegie Stage Comparisons (https://embryology.med.unsw.edu.au/; and references within), using the presence of gross morphological characteristics, such as the first appearance of somites, development of the heart, closure of the posterior neuropore (neural tube) and embryo turning, but specifically did not measure events with obvious heterochrony including appearance of the limb primordia. Stage‐matched Carnegie stage (CS) comparisons are shown visually in Figure 6 and extended comparisons are outlined in Table 1.

**TABLE 1:** Embryonic stage‐match estimations between amniotes.

Taxa		Embryonic day (E)																
Clade	Species	8	9	10	11	12	13	14	15	16	17	18	19	20	21	22	23	Carnegie Stage
Eutheria	Human	17–19	20	22	24	28	30	33	36	40	42	44	48	52	54	55	58	Day
	Baboon			25	27	28	29	30	31	33	35	37	39	41	43	45	47	
	Rhesus Monkey			22	25	28	29	30	32	34	36	37	38	40	42	44	46	
	Mouse	6	7	8	9	10	11	11.5	12	12.5	13	13.5	14	14.5	15	15.5	16	
	Rat			11	11.5	12	12.5	13	13.5	14	14.5	15	15.5	16	16.5	17	17.5	
	Chinese Hamster			10.5	11	11.5	12	12.5	13	13.5	14	14.5	15	15.5	16	16.5	17	
	Guinea Pig			15	15.5	17	18	19	20	21	22	23	24	25	26	27	29	
	Rabbit			8.5	9.5	10.5	11	12	12.5	13.5	14	14.5	15.5	16	16.5	17	18	
	Sheep			16	17.5	18.5	19.5	20.5	22	23	24.5	25.5	27.5	29.5	30	33		
	Pig			15	16	17	18	19	20.5	21.5	23	24	25.5	27.5	29	30.5	32.5	
Marsupial	Dunnart	E10.25	E10.5	E10.75	E11	E11.5	E12	E12.5	12.75	E13	E13.5	P0‐3	P4‐7	P8‐11	P12‐16	P17‐19	P20‐23	
Aves	Chicken	1	1.25	1.5	2	2.25	2.5	3	3.25	3.75	4.75	5.5	6.25	7.25	7.75	8.5	10	

*Note*: Carnegie stage comparisons were taken from UNSW Embryology and modified to include matched dunnart stages.

Dunnart organogenesis begins at embryonic Day 10 through the appearance of the planar primitive streak embryo, characteristic of the human CS8 or approximate mouse E6 (Thieler stage, TS9) or chicken E1 (Hamburger–Hamilton stage, HH3–4) embryo. With this, we estimated stage‐matched embryos from CS8 to CS18. CS10 embryos possess headfolds and the first presence of somites. We therefore estimated that the stage 24 dunnart (E11) was roughly equivalent to a CS10 human embryo, and stage‐matched TS12 mouse and HH12 chicken embryo (Table 1). However, notably stage 24 dunnart embryos display considerable accelerated development of the facial prominences and forelimbs, which already extend from the elongated embryo at this early stage of development. General development of Dunnart embryos was matched to subsequent Carnegie stages based on gross morphological characteristics (Figure 6, Table 1). However, the accelerated craniofacial and limb development become more obvious, whereby approximately CS17–CS20 (stage 31–33 and immediately following birth) the dunnart maxillary and mandibular prominences have elongated into defined jaws, and the forelimbs possess defined digits and musculature, while other amniotes retain more rudimentary features. These comparisons highlight that the onset of marsupial developmental heterochrony is rapid, first appearing between CS8–10 and persisting throughout subsequent stages. Importantly, these contrasts lay future foundations for comparative molecular and genetic studies into the origins of developmental heterochrony and plasticity between amniotes.

## 3 DISCUSSION

The fat‐tailed dunnart (*S*.*crassicaudata*) is a small, mouse‐like dasyurid marsupial that breeds successfully in captivity year‐round. The dunnart has one of the shortest gestation and organogenesis periods of any mammal,^25^, ^26^ pushing the known limits of mammalian development. Compared with equivalent‐sized eutherians (i.e., rodents), this represents one of the most extreme examples of heterochrony within established laboratory models. These characteristics make it a valuable tool to study key processes underlying mammalian development, and further understand the defining characteristics of marsupials. To capitalize on this model, we established robust methods for pregnancy monitoring and timed embryo collection and presented a high‐resolution characterization of the embryology and prenatal development of the dunnart from cleavage to birth. Furthermore, stage‐matched embryological estimates with other amniote model species (human, mouse, chicken, etc.) were generated to open the door for future comparative studies. The dunnart shows extraordinary acceleration of head and forelimb development, emphasizing the extreme developmental heterochrony observed in marsupials and highlighting their utility for mammalian evolution. Combined with recent reconstructions of Dunnart postnatal growth and development,^25^, ^26^ this work provides essential resources for utilizing the Dunnart as a new and emerging mammalian laboratory model.^25^, ^26^, ^50^

Dasyurid marsupials (order Dasyuromorphia) possess a more rapid timetable of development than other marsupials, giving birth to some of the most altricial young of all mammals. Classed as G1 or “ultra‐altricial”,^29^, ^60^ dasyurids are usually born after a short gestation of between 11 and 20 days, just a few days after initiation of gastrulation, and can weigh as little as 10 mg at birth.^28^, ^30^, ^60^ While previous studies have detailed aspects of the embryonic growth and development of *S*. *crassicaudata*,^42^, ^45^, ^46^, ^47^ little information existed for routine and reliable generation of timed embryos. We therefore applied monitoring methods established in *S*. *macroura* ^37^, ^61^, ^62^ and refined these to produce a universal reproductive profile and formula to predict staged embryos. In *S*. *macroura*, the day of ovulation coincides with a fall in body weight, the presence of cornified epithelial cells, and appearance of polymorphonuclear lymphocytes, and the occasional presence of sperm in the urine.^37^, ^61^, ^62^ However, we found that monitoring daily weight fluctuations, with or without added urine cytology (Figure 1), provides a minimally invasive approach for reliable prediction of pregnancy, leading to a >75% success rate for embryos. Timed collection of pregnant females yielded up to 17 preimplantation embryos, and up to 10 fetuses before birth per individual animal (Figure 2C). Though slightly more involved than observation of a post‐copulatory vaginal plug in mice, this method provides a cheaper and high throughput alternative to camera monitoring methods necessary for *Monodelphis*.^34^ Application of these methods produced a complete characterization of *S*.*crassicaudata* embryogenesis from cleavage to birth (Figures 4 and 5), and detailed descriptions for the visualization and collection of the embryos from within the dunnart uterus.

To further improve the utility of our series for future comparative investigations, we sought to establish stage‐matched embryonic comparisons with other amniote models. Previous comparisons of neurological development (brain and eye) between dunnart and mouse suggest the newborn dunnart aligns with a CS18 embryo (or mouse E13.5). Whereas gross observations of features within the head, body, limbs, and tail suggest the newborn dunnart morphologically resembles a CS20 embryo,^58^ where each species shows fused facial prominences, limbs with demarcated digits, closed ventral body wall, and extended tail. Owing to their short gestation and functional constraints at birth,^14^, ^22^, ^29^, ^32^ the marsupial neonate displays accelerated development of the forelimbs, craniofacial bone, and oral musculature,^16^ but delayed development and maturation of the eyes and brain,^26^, ^27^ making precise stage‐matched comparisons difficult. As such, while the dunnart shows a combination of advanced and delayed features at birth, overall, the newborn dunnart more closely aligns with embryonic stages across birds and mammals than any postnatal stage. However, additional embryonic comparisons were needed to establish a complete matched series.

The dunnart primitive streak embryo was set to match CS8, through the first formation of the primitive streak, allowing the estimation of stage‐matched embryos from CS8 to CS18. Remarkably, as dunnart organogenesis proceeds there was accelerated formation of the craniofacial and limb primordia at early equivalent developmental stages, in accordance with their functional demands at birth. By CS10 (mouse E10.75, chicken HH12), denoting the first few somite‐pair embryo, stage‐matched dunnart embryos (stage 24) already showed patterning and outgrowth of the frontonasal, maxillary, and mandibular processes and forelimb ridges. This is approximately two stages earlier than the onset of these developmental events in human, mouse, or chicken, which each occur at ~CS12 (mouse E10.5, chicken HH18). Furthermore, by CS17 (mouse E13, chicken HH24), the dunnart embryo has well‐developed forelimbs with demarcated digits, and elongated jaw primordia, showing considerable acceleration compared to other amniotes. Studies in the opossum have revealed that marsupials indeed possess heterochronic acceleration of the neural plate, neural crest, and craniofacial development,^21^, ^22^ as well as early specification and formation and outgrowth of the forelimbs,^20^ showcasing the extraordinary marsupial‐specific heterochronic patterns relative to other amniotes. Furthermore, due to their shorter gestation, these events appear to occur even more rapidly in the dunnart, providing an excellent system to examine how heterochronic outcomes are controlled during development.

Few studies have interrogated the molecular mechanisms underlying limb and craniofacial heterochrony in marsupials, presenting a large gap in our understanding of these processes. Comparative investigations in *Monodelphis* have revealed early activation of key fate‐specific genes during craniofacial (*SOX9*) and limb (*TBX5*, *FGF10*, *FGF8*, and *SHH*) development^20^, ^31^, ^63^, ^64^; though how these genes and their respective networks are differentially regulated to drive these distinct morphological outcomes is unclear.^41^ While recent attempts have begun to generate comparative embryological, genomic, and single‐cell investigations to model differences in development between different vertebrates,^6^, ^39^, ^65^, ^66^ such comparisons are still in their infancy. This presents unique future opportunities to capitalize on the dunnart as a model species with which to comparatively interrogate how heterochrony and developmental plasticity are regulated at the cellular and molecular levels.

The current global extinction crisis emphasizes our need to comprehensively understand mammalian development and to develop tools to conserve its biodiversity. This study addresses the need for accessible marsupial models by establishing the fat‐tailed dunnart as a laboratory model for comparative embryology and genomics, and we provide robust methods for animal husbandry, reproductive monitoring, and timed embryo collection to support the use of this model organism. Comparisons with other species highlight the key features of marsupial development, opening avenues for future comparative studies to deepen our understanding of mammalian development and diversity. With the establishment of the dunnart as a marsupial laboratory model, the embryological toolbox for developing foundational knowledge and exploring conservation strategies has become more thoroughly equipped.

### 3.1 Experimental procedures

#### 3.1.1 Animal care, husbandry, and monitoring

Fat‐tailed dunnarts (*Sminthopsis crassicaudata*) are small, carnivorous marsupials from the family Dasyuridae, which are easily kept in captivity. Dunnarts were housed and bred within a dedicated facility within the School of Biosciences. Animal husbandry and cycle monitoring were adapted from methods established in *Sminthopsis macroura*.^37^, ^61^ Best practices for the housing, care, and mating of dunnarts are detailed in a companion study,^50^ but summarized below. The oestrus cycle of the dunnart is ~31 days,^67^ with a gestation of ~14 days.^29^ Dunnarts give birth to up to 10 pouches young, aligning with number of nipples, with an average of 7 young per litter. Upon removal or loss of pouch young, females will ovulate again up to 14 days later^68^ depending on the age of the pouch young, which can be utilized to time successive matings. Matings were set up within a simulated “summer” room (temperature 25°C; light cycle 16 h light:8 h dark) mimicking natural summer conditions to encourage year‐round mating and given food and water ad libitum.

Female and male dunnarts were allocated into mating cages in a 3:1 or 2:1 ratio of females to males. Monitoring of female dunnarts began immediately upon allocation into a mating pair, with monitoring occurring generally between 7:00 and 8:30 am on weekdays, and occasionally on weekends when pregnancy was suspected. Early monitoring was beneficial both in increasing the likelihood of sperm detection (assuming dunnarts ovulate early in the morning)^52^ as well as ensuring dunnarts were weighed prior to feeding. Female dunnarts were weighed daily, and their urine was collected and examined under 4× and 10× magnification for the presence of sperm. Weight increase was used as the primary indicator of pregnancy, while sperm presence was treated as a secondary indicator. Routine monitoring was used to estimate both pregnancy and embryo stages for collection and was slowly adapted and improved through obtaining more data from collections of pregnant females.

#### 3.1.2 Establishing reliable methods for timed embryo collections

Weight changes have been related to stages in the cycle and the stages of pregnancy in *A*. *agilis* ^36^ and *S*. *macroura*.^37^, ^51^ During dunnart (*S*. *crassicaudata*) cycling, there are considerable weight fluctuations (in grams) which were considered to likely be a strong predictor of pregnancy (proportional to body weight, from average weight ~16 g). Notably, these included a subtle weight increase leading into oestrus, followed by a sharp ~2 g weight drop during ovulation. If fertilization occurred, a steady ~3–4 g weight increase ensued over the following 14 days of the pregnancy cycle. Females were allocated to timed matings at random points in their cycle (in these cases we only used weight increase to determine pregnancy), and pregnant females that gave birth were used to assist in calibration of the weight cycle and predict ovulation. These females had their pouch young removed between 0 and 65 days after birth, then were monitored for a weight dip (~8–12 days) before being allocated to a mating pair. Placing females with males within 1‐day preceding ovulation was found to be more effective in generating a pregnancy, though males would typically only mate with one female in cases where two synchronized females (i.e., siblings or animals displaying similar weight fluctuations) were placed in the same breeding cage. In instances where females did not fall pregnant, their weight changes underwent similar fluctuations to that of pregnant individuals, albeit with a much lower and gradual weight increase of 1–2 g, rather than 3–4 g. A standardized plot of pregnant dunnarts was generated to better inform subsequent collections. To do this, weight change between different‐sized and aged animals was normalized by taking the percentage daily weight change per animal. This was calculated by taking the average pre‐ovulation (anoestrous) weight, taken to be the average *base weight* of an animal, then expressing the daily weight across the cycle as a percentage of the base weight – using the formula: ([Daily weight – Base weight]/Base weight) × 100 (Figure 1A).

In conjunction with weight change, urine cytology has additionally been shown to be a strong predictor of estrous in *A*. *agilis* ^36^ *and S*. *macroura*.^37^, ^51^ As such, urogenital lavage was performed to identify the presence of enucleated cornified epithelial cells (CEC), nucleated epithelial cells (NEC), and leukocytes to predict the onset of estrous. Four adult female dunnarts, aged between 119 and 124 days old, were monitored daily with urogenital lavage collection over a period of 3.5 months. Female dunnarts were gently picked up with one hand and 20 μl of phosphate‐buffered saline (PBS; Thermofisher) was placed at the opening of the urogenital canal. Fluid was expelled 1–2 times to flush the canal and drawn back into the pipette tip. Collected fluid was placed on a glass slide and left to dry completely on a heat block set to 40°C. For cytological assessment, slides were stained with 0.1% crystal violet (Sigma) for 3 min, followed by washing in running water for 1 min, then mounted under a coverslip with the addition of 15 μl of glycerol. Slides were examined using a Motic microscope (Motic Scientific, San Antonio, TX), and the numbers of CEC, NEC, and leukocytes were counted. Proportions of each of the three cell types were manually counted twice on two different fields under 200× magnification, with one slide processed per animal. The average number of the two counts was used for data analysis using GraphPad Prism (Version 10; Boston, MA).

#### 3.1.3 Ultrasound and anesthesia

Ultrasound exams were conducted using an MyLab Sigma portable ultrasound unit (Esaote, Genoa, Italy) with a 22 MHz linear probe with images produced using the onboard software. Dunnart anesthesia was achieved using the methods described by Paolino et al.^48^ Briefly, pregnant female dunnarts are placed in a sealed chamber and delivered 5% isoflurane in medical‐grade oxygen at a flow rate of 200 ml/kg/min. Once unconscious, females are transferred onto a heated stage and maintained by supply of 3% isoflurane through a custom small animal mask. The hair surrounding the pouch area was shaved using a small set of electric clippers before the application of ultrasound gel. To reduce the burden of anesthesia on animals, examinations lasted no longer than 5 min.

#### 3.1.4 Dissection and embryo collection

Pregnant females (those showing successive days of weight increase after the ovulation dip) were humanely killed via cervical dislocation. The abdominal cavity was opened, and the reproductive tract was removed and washed in sterile PBS. Uteri were dissected in sterile PBS, and excess connective tissue, ovarian material, and fat were removed. In clean PBS, uteri were then bisected by a medial incision to allow pre‐implantation embryos (stage <26) to freely roll out into the dish. Implanted embryos were carefully cut out with their membranes intact. All embryos were transferred with a wide‐bore transfer pipette immediately into 4% PFA (ProSciTech) and fixed for up to 72 h at 4°C. Fresh or fixed embryos were washed in fresh PBS and imaged on a SZX16 Stereo dissecting microscope with an SDF PLAPO 1XPF Objective (Olympus Life Sciences), or SteREO Discovery V12 with an Achromat 0.63× Objective (Zeiss), depending on developmental stage and size. The number of pregnant females, which contributed to specific embryo stages, are summarized in Table 2.

**TABLE 2:** Number of pregnancies collected per day of embryonic development.

Gestation day	Day 9	Day 10	Day 11	Day 12	Day 13
Stage range	< St.19	St.19–23	St.24–27	St.28–30	St.31–33
Number of animals collected	6	13	5	3	6

*Note*: Summary of the number of female animal collections at each day of gestation which contributed to specific‐staged embryos.

#### 3.1.5 Microscopy

Fluorescence microscopy was utilized to better visualize the anatomy and morphology of early embryos. Fixed embryos were stained with the nuclear marker DAPI (1:1000) to visualize the embryo surface, and early neurulation stage embryos were additionally stained for either E‐cadherin (1:500; Abcam AB76055) or F‐actin (Phalloidin‐iFluor 488, 1:1000; Abcam AB176753) to highlight the presence of different tissue types. Whole‐mount embryos were imaged on a Nikon A1R confocal microscope on a 10× objective using NIS‐Elements software and processed using Fiji.^69^

## FUNDING INFORMATION

This research was conducted under research funding through Australian Research Council DP210102645 and DP160103683 to Andrew J. Pask, UoM ECR grant TP605149 to Axel H. Newton, generous philanthropic funding from the Wilson Family trust, and industry funding through Colossal BioSciences.

## CONFLICT OF INTEREST STATEMENT

The authors do not declare any conflicts of interest.

## Supporting information


**Table S1.** Raw and transformed cycle weight data from monitored female dunnarts. Individual dunnarts were monitored daily for weight and were aligned based on either stage of collected embryos or birth of pouch young. Conditional formatting was applied to visualize weight decrease/increases. Average weight with standard deviation is shown for individual and all animals. Normalized daily weight changes were calculated using the formula ((Daily weight – Base weight)/Base weight) × 100. Where no anoestrous data was present, profile could not be determined for that animal. Normalized data are shown in table. Day 14 of gestation was noted as day of birth.
